# Remarks on Mr. Harrold's Case of Paralysis

**Published:** 1813-06

**Authors:** 


### *To the Editors of the Medical and Physical Journal.*

"Varietas remediorum ignorantiæ filia est."—VERULAM.

GENTLEMEN,

IN the Number of your Journal for December, a case of Paralysis is inserted, by Mr. Harrold, who solicits the remarks of Dr. Cheyne concerning it. The silence of that gentleman makes me anxious that Mr. H.'s request should not be ungratified, as there appears to me much reason for remarks both on the history and treatment.

Passing over Mr. H.'s opinion concerning elastic and compressible vapour pervading the ventricles, and interposing between the convolutions of the brain; and that palsy and apoplexy, though relations, are not the same individuals, I shall proceed to the case and practice.

"A carpenter, aged 52, (for we have nothing to do with his punctuality in business, &c.) having for some days had obscure feelings of indisposition, with a sallowness of complexion, (query of a sallow complexion?) was suddenly seized, on the 3d of March, with excessive giddiness, as he sat at his desk, and hemiplegia of the left side." Mr. H. was sent for immediately, but, before he could arrive, another practitioner had administered some eau d'luce. "The pulse was feeble, irregular, and indistinct, and he complained of an icy coldness of the affected side." To "rouse" the patient, as he says, from this torpid state, hot and strong brandy and water was given. Vomiting (fortunately) soon came on, and, upon the stomach being emptied, the patient exclaimed, "Thank God, I have got the use of my arm and leg again!" "His memory was a good deal impaired, but this was not a new circumstance." Formerly he had suffered from rheumatism, and irregular gout affecting his stomach, but not for some years.

Was ever history so imperfect? What is of no consequence, is told: what is of consequence to know, is omitted. Had, for example, his fulness of habit been mentioned, instead of his regularity of habits—the state of the bowels, of the stomach, of the tongue, of his appetite, his digestion, his sleep, some accurate judgment might have been formed, whether the principal and primary seat of the disease was in the head or the stomach, though there can be little doubt but that it was in the latter.

The sallowness is spoken of so indistinctly, as to render it doubtful whether it was usual, or only of the date of his present illness. Nothing is said of the fluid vomited, which might have proved that some improper or indigestible food laid laid in the stomach. Even the position of sitting long at a desk, has in many people produced convulsions and fainting to a violent degree, and, for any thing we are told, Mr. G. might have had his dinner, and been pent up in a warm and close room, thereby increasing his predisposition.

We are not told in what manner he had felt indisposed during the preceding days, whether or not the bowels *were punctual*, or whether head-ach was a precursor; nor is difficulty of deglutition mentioned as having ensued. The feeble, irregular, and indistinct pulse, rendered it more probable that only the stomach was affected, and pointed out its speedy evacuation to be requisite, by warm water, or an emetic. It has not unfrequently happened to me, to see children, and even adults, where paralysis was produced by no other cause, which was diminished, made intermittent, or removed, according to the mode and frequency of employing evacuants. In what manner a disordered state of the stomach and bowels act upon the brain and nerves, I am unable to say; but the fact is certain, though no organic disease be produced by it in the brain itself.

Let us next examine the propriety of the practice, by the help of such symptoms as are enumerated. The first practitioner administered eau d'luce, and Mr. H. hot brandy and water, under the impression, too, that the disease proceeded from a fullness of blood! Though it is difficult to say which practice was the most injudicious, it is easy to prove they were both highly so, for no plan can be so injurious as that of "*rousing*" which they adopted. Indeed, had the paralysis proceeded from the rupture of a blood-vessel in the brain, which is commonly the case, was increasing the circulation likely to relieve the pressure, or diminish the rupture?

Fortunately for the patient, and contrary to the sagacious intentions of his advisers, vomiting came on, the stomach being already disordered, and unable to bear any farther accumulation, which had been the cause of the complaint. To those who make any other use of the indications by the pulse, than to ascertain the velocity of the circulation, nothing could have better foretold that vomiting would ensue, than its feebleness, irregularity, and indistinctness. The complete evacuation of the stomach, by removing the cause of the paralysis, again restored the patient to the use of his limbs.

After vomiting, a purgative was very properly administered. But a blister between the shoulders, a camphor liniment to the limbs, and cordial confection with carbonate of ammonia every six hours—was useless and painful—was unnecessary—

necessary—was hurtful! We hear nothing of the patient during the 4th, nor whether his purgative had acted, or if it had, what was the nature of the evacuations. But, on the evening of the 6th, one grain!!! of calomel was given, and the purgative to be repeated in the morning. The other remedies, viz. carbonate of ammonia, and cordial confection, to be continued. On the 7th, no mention is made of the bowels, but the patient complained of some oppression at his stomach (was it wonderful?) to relieve which a *cummin plaster* was applied across it, an infusion of gentian joined to the ammonia internally. From the admixture of purges, gentian, ammonia, &c. and, if we may judge from this specimen of judicious treatment, probably pork, porter, port-wine, and suet dumplings, was it unnatural that the stomach should complain, and be oppressed, till soothed by the anodyne powers of a *cummin plaster!* The 8th is a blank, but on the 9th the purgative was ordered to be repeated occasionally: the calomel (gr. 1) having affected his bowels rather too powerfully, was not repeated! Had we been told what was the nature of the former purgatives, and what effect they produced, we might have been better able to judge how far *one grain of calomel* could produce this new and astonishing effect. Let the whole tribe of domestic medicine-givers, and three-fourths of the practitioners in England, who may be said *to feed* their patients on calomel, tremble at the devastation their ignorance of its effects must produce! On the 11th he had another attack, not quite so severe as the first, but depriving him of the use of his *right* arm. "Imitating nature, in the previous attack," says Mr. H., "I gave an emetic of the sulphate of zinc; it operated, but did not seem to clear the stomach. After a few minutes, the purgative that had been omitted in the morning was given, which completely evacuated the stomach upwards, with the instantaneous restoration of the right arm." The nature of the purgative, and the quantity and contents of the stomach, are again withheld from us.

To continue—"This day he was visited by Dr. Smith, of Hertford, who advised blisters behind the ears, a purgative in a different form, and volatile guaiacum, to be added to the former medicines. On the 12th, a more active purgative was given, the last not being sufficiently so. On the 13th, the blister between the shoulders was renewed, and ordered to be kept open, and 4 grains of the pil. hydrarg. to be taken night and morning, with cascarilla, and volatile tincture of guaiacum, every eighth hour. The loaded state of the urine, with the pink sediment, denoted hepatic affection, which gradually yielded to the mercurial pill."

"I," says

"I," (says Mr. H.,) "particularly insisted upon the discontinuance of *tea*, and which, in his ordinary state of health, if he *exceeded* his usual quantity at breakfast, frequently occasioned full vomiting." Mr. H. first reasons, that vomiting cures his patient. 2dly, Because *tea* causes vomiting, he forbids its use. 3dly, If taken in usual quantity, it does not cause vomiting. 4thly, Because it may be taken in immoderate quantity, and cause vomiting, it is to be discontinued. Now, excepting this good carpenter's *want of punctuality* to restrain himself to a due portion of tea, which probably comforted him, as it does most people, and was the least injurious beverage he took, I can see no objection to his use of it, but much to the mixtures forced upon his *delicate stomach*, in spite of all the loathing he must have felt. "On the 20th a seton was placed in the neck, *as a permanent remedy*." A permanent remedy, for what?—for indigestion! On the 22d an opiate was prescribed for him at bed-time, to the use of which, says Mr. H., *I was reluctantly driven*, in consequence of his having suffered extreme pain, not only in the seton since its insertion, but also in the extremity of the fourth finger of the right hand, extending up the arm, which had deprived him of sleep ever since the 11th; that is to say, he had passed ten nights without sleep, from pain, before Mr. H.'s *reluctance* to grant him ease could be conquered. "The opiate procured ease and sleep, without any ill effect whatever." Is this wonderful?

I shall pass over the logical account of the separation of the nail, "except at its root and sides, the morbid sensibility of the part, till *every particle* of the nail, *root and sides*, was removed," &c.

This plan of treatment was pursued till the end of April. He had a slight attack on the 9th of June, occasioned by fatigue and anxiety, *and drinking several cups of tea.* An emetic relieved him, and he has since gradually improved in health. The seton keeps discharging.

As nineteen cases of paralysis in twenty affecting one side proceed from disease within the head, and that, most frequently, from a rupture of a blood-vessel, is it not lamentable, that a fact so universally known and described, could be so little understood, as in the example before us; so that people practising medicine, with the intention too of benefiting their patients, should err so egregiously, in spite of professional authority, and the dictates of common sense, as to endeavour to remove a complaint by increasing what they believed to be its causes? Who ever attempted to recover a drowned person by pouring more water upon him? Yet the one is not more unreasonable than the other. It is not less gross, in a case wherein every possible indication by symptoms proved the stomach to be the seat of the disorder, not to apply the remedies proper to relieve it, and these only.

Having already detailed what was done, I shall merely recapitulate how much of it was unnecessary, and even hurtful.

The eau de luce, and hot brandy and water, had it been from a ruptured blood-vessel, might have rendered the injury irrecoverable. The blister to the shoulders, the liniment, and the ammonia, were highly injudicious. It was extremely negligent not to examine the evacuations, or detail the kind and quantity of the purgatives, which seem to have been given in a vague undetermined manner; and Mr. H. might as soon make me believe that he saw a man fly, after swallowing a grain of gunpowder, as, contrary to all experience and all information, credit that one grain of calomel produced such effects upon the bowels; though attributing to A. what C. might do but B. did, is the most common of all medical reasoning. Besides, who ever heard of a cummin plaster being applied to relieve a pain in the stomach, produced by its injurious contents? The second attack, and the vomiting, again showed from whence the mischief originated.

As for the blisters behind the ears, they had no more connection with the cure of the complaint, than cutting his ears off would have had; and, though no fault could be urged to the pil. hydrarg., why was the volatile tinct. of guaiacum, so disgusting and hurtful a medicine, in this complaint, given, and even added to the others? The *permanent remedy* of the seton, and the ridiculous fear of giving opium, have been already mentioned.

After the painful duty of pointing out what ought not to have been done, it is but just to state what I should have done. On being called to Mr. G., I should have given him warm water, chamomile tea, or an emetic, to evacuate the stomach—an enema to empty the intestines—and followed that by an active cathartic. I should have ordered Mr. G. to bed, increased the warmth of his extremities, and probably even have allowed him freely of the dangerous liquor called *tea*. I should have given the pil. hydrarg. or pil. aloes every night, and some bitter infusion with salts in the day, to act constantly but gently upon the bowels; have examined the evacuations until they became healthy, and the tongue clean; and also have regulated his diet and exercise. This plan would soon have restored Mr. G. to health, and probably without his having had any second attack, and at least without without blisters, setons, cordial conf. ammonia, guaiacum, or even a cummin plaster.

Without any knowledge of the parties, but by name, through the medium of your Journal, I cannot be accused of personality in the freedom of my strictures on the above case; nor do I conceal myself from fear, but prudence, as the name can be of no moment where the doctrine, and not the person, is censured. My only wish is to see the profession more respectable, and its followers more respected; to suppress that rage for uncommon cases, which is now so prevalent; and that silly vanity of the multitude, who risk reputation, and even esteem, for the empty conceit of seeing their name in print. Let my opinions be proved wrong, and if the attack be just, I shall defend them.—Nunquam enim noster est animus in ullius hominis vel medici famam injurium esse: errantibus tamen monstranda est via, salebræ in praxi deligendæ, et effusa menti caligo abstergenda est.

I am, Gentlemen,

Your obedient Servant,

A PRACTICAL PHYSICIAN.

P. S. I purpose to send a few remarks upon Dr. Buxton's regulated temperature, for next month; wherein I shall prove it a medical *Whim-wham!*

*London, May* 1, 1813.

